# Motor cortical circuits contribute to crossed facilitation of trunk muscles induced by rhythmic arm movement

**DOI:** 10.1038/s41598-020-74005-z

**Published:** 2020-10-13

**Authors:** Shin-Yi Chiou, Laura Morris, Weidong Gou, Emma Alexander, Eliot Gay

**Affiliations:** 1 School of Sport, Exercise, Rehabilitation Sciences, University of Birmingham, Edgbaston, Birmingham, B15 2TT UK; 2 Centre for Human Brain Health, University of Birmingham, Birmingham, UK

**Keywords:** Neuroscience, Motor control, Motor cortex

## Abstract

Training of one limb improves performance of the contralateral, untrained limb, a phenomenon known as cross transfer. It has been used for rehabilitation interventions, i.e. mirror therapy, in people with neurologic disorders. However, it remains unknown whether training of the upper limb can induce the cross-transfer effect to the trunk muscles. Using transcranial magnetic stimulation over the primary motor cortex (M1) we examined motor evoked potentials (MEPs) in the contralateral erector spinae (ES) muscle before and after 30 min of unilateral arm cycling in healthy volunteers. ES MEPs were increased after the arm cycling. To understand the origin of this facilitatory effect, we examined short-interval intracrotical inhibition (SICI) and cervicomedullary MEPs (CMEPs) in neural populations controlling in the ES muscle. Notably, SICI reduced after the arm cycling, while CMEPs remained the same. Using bilateral transcranial direct current stimulation (tDCS) in conjunction with 20 min of the arm cycling, the amplitude of ES MEPs increased to a similar extent as with 30 min of the arm cycling alone. These findings demonstrate that a single session of unilateral arm cycling induces short-term plasticity in corticospinal projections to the trunk muscle in healthy humans. The changes are likely driven by cortical mechanisms.

## Introduction

Trunk muscles are activated prior to or concurrent with voluntary movements of upper limbs to provide stability to the body, allowing for efficient function of the upper limbs^1–3^. For example, reaching for objects beyond the arm’s length and tasks requiring rhythmic arm movement (e.g. arm cycling and walking) involve activation of trunk muscles^4–7^. When trunk control is impaired, movements of the upper limbs are often altered, thereby limiting their use^6,8^. Despite evidence of functional interactions between the arm and trunk muscles, neural mechanisms underpinning the functional interactions between upper limbs and the trunk remain largely unknown.

Training of one limb through task repetition improves the performance with the contralateral, untrained limb^9–12^, a phenomenon known as cross transfer. For instance, increased peak acceleration was observed in both left and right hands performing index finger abduction after the unilateral training of the finger abduction of the right hand^10,13^. The mechanisms contributing to the cross transfer between the upper limbs are likely from bilateral interactions of the primary motor cortices (M1s)^14^. Previous work has shown a decrease in interhemispheric inhibition from the trained M1 to the untrained M1 after the unilateral training^15^. Furthermore, research using transcranial direct current stimulation (tDCS) over the M1 contralateral to the untrained muscle demonstrated enhancement of the cross-transfer effect in the untrained muscle further supporting this cortical mechanism^9,16,17^. Growing evidence suggests that the physiological pathways modulating upper-limb and trunk muscles interact^3,18–20^. However, it remains unclear whether training of the arms can induce plasticity in the neural pathways projecting to the trunk muscles, resulting in improved trunk function. Research has shown that reductions in GABAa-mediated intracortical inhibition, i.e. short-interval intracortical inhibition (SICI), contributing to plasticity in the M1s are related to the cross-transfer effect and motor skill acquisition^21,22^. Our previous study found that voluntary contractions of biceps brachii (BB) and triceps brachii (TB) reduced SICI and increased corticospinal drive to the erector spinae (ES) muscle in the lower thoracic region^19^. This highlights the potential of improving performance of the ES muscle by employing arm movement. Since many patients with neurologic disorders, such as stroke^23^ and spinal cord injury^24^, have impaired trunk control, understanding if arm-training can influence function of the trunk muscles will lead to development of interventions for trunk rehabilitation. For instance, arm ergometer exercise requires minimal supervision and inexpensive equipment, therefore providing a suitable patient-led rehabilitation technique in the community.

**Figure 1:**
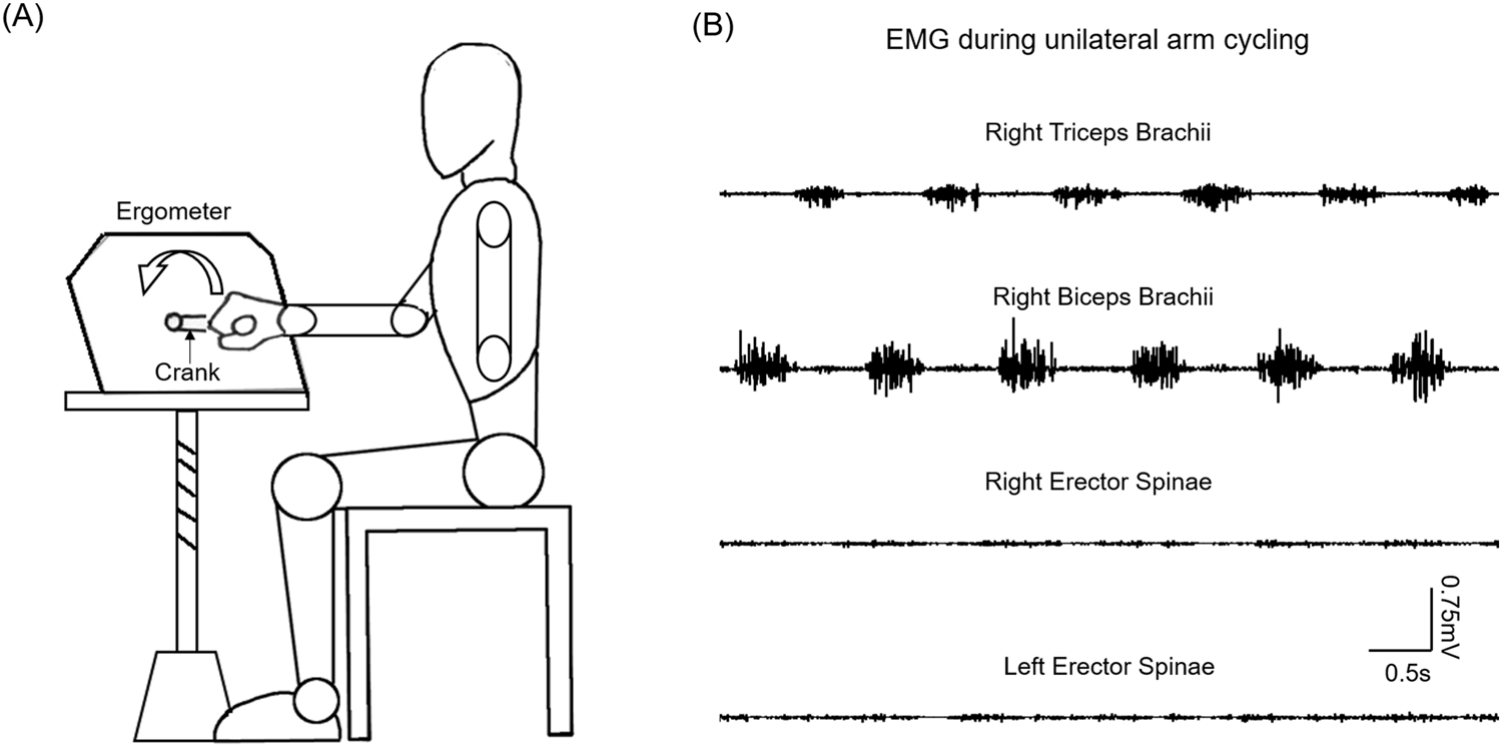
Experimental setup. (**A**) Schematic illustration of the setup of unilateral arm cycling. (**B**) Raw electromyography (EMG) activity recorded from biceps brachii and triceps brachii muscles of the dominant arm performing the arm cycling, and from bilateral erector spinae muscles.

Previous studies have shown changes in M1 excitability projecting to a trunk muscle that is not primarily involved in upper-limb movements. For example, interhemispheric inhibition is present in the ES muscle located at the first lumbar vertebra during voluntary contractions of anterior deltoid (AD)^25^. In addition, studies have shown the contribution of the M1 to postural adjustments of the ES muscles located at the lower thoracic region during bilateral shoulder movements^3,26^. Thus, we hypothesised that arm training increases corticospinal projections to the trunk muscles, which are not the prime movers used in the training, and the mechanisms are likely involved cortical circuits within the M1. To test this hypothesis, we examined corticospinal excitability, intracortical inhibition and spinal excitability in the ES muscle using TMS and electrical stimulation prior to and following unilateral arm cycling (Fig. 1). Based on previous studies using tDCS to examine cortical mechanisms, we also tested the effect of combined bilateral tDCS and the arm cycling on changes in the size of ES MEP in healthy participants.

## Results

### Moto evoked potentials (MEPs) in the ES muscle increase after the arm cycling

**Figure 2:**
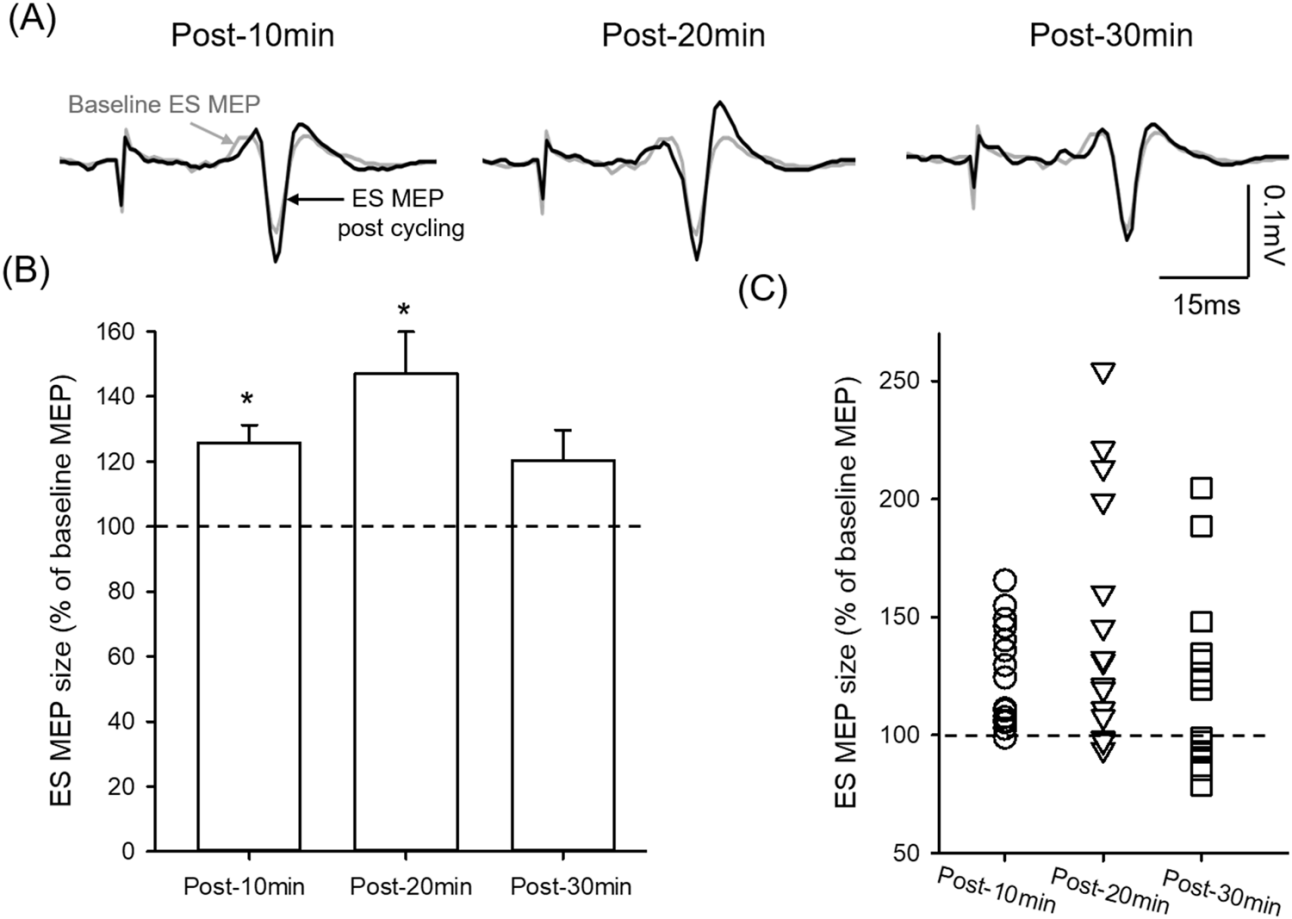
Motor evoked potentials (MEPs) in the erector spinae (ES) muscle. (**A**) MEP traces recorded from the ES muscle of a representative participant. Traces show the average of 10 MEPs in the ES muscle before (grey graces) and after (black traces) 30 min of unilateral arm cycling. (**B**) Group data (n = 15) showing ES MEPs after the arm cycling. The abscissa shows the time points measured after cessation of the arm cycling and the ordinate shows the size of ES MEP (as a % of the ES MEP obtained prior to the arm cycling, called baseline). The horizontal dashed line represents the size of the ES MEP at baseline. Note that the size of ES MEP increases at 10 and 20 min after the arm cycling compared with baseline and returns to the same as baseline at 30 min after the arm cycling. (**C**) Individual data showing that majority of participants demonstrate increases in ES MEP at 10 and 20 min after cessation of the arm cycling. Error bars indicate the standard error of the mean (SEM). *p < 0.05, comparison between post-exercise and baseline.

Figure 2A illustrates traces of averaged MEPs in the ES muscle from a representative participant. Note that the size of MEP in the ES muscle increased after the arm cycling for up to 20 min. Results demonstrated an effect of TIME on the size of MEP in the ES muscle (F_3,42_ = 6.18; p = 0.001; n = 15). Post hoc tests showed that the amplitude of ES MEP increased at 10 min (125.85 ± 21.60% baseline MEP; p = 0.04), and 20 min (147.12 ± 51.21%; p = 0.001) following the arm cycling compared with baseline (Fig. 2B). There was no difference in the size of ES MEP between 10 and 20 min after the cycling (p = 0.23). Note that majority of participants show increased MEPs in ES at 10 and 20 min after the cessation of the arm cycling compared with baseline (Fig. 2C). There was no difference in ES MEP at 30 min after the arm cycling (120.40 ± 37.29%; p = 0.104) compared with baseline. There was no difference in background EMG in ES across all time points (F_4,42_ = 2.16; p = 0.12).

### Short-interval intracortical inhibition (SICI) in the ES muscle decreases after the arm cycling

**Figure 3:**
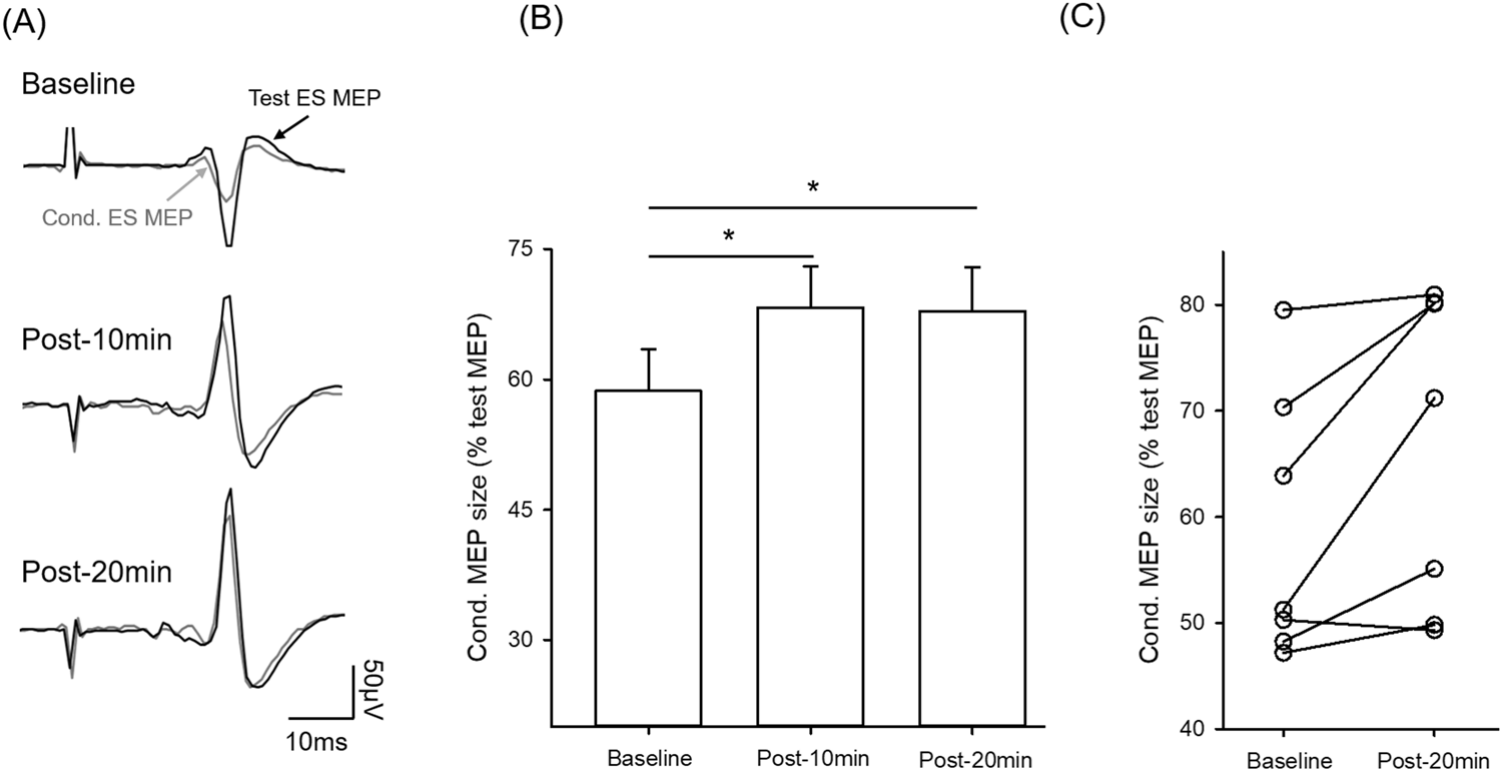
Short-interval intracortical inhibition (SICI) in the erector spinae (ES) muscle. (**A**) SICI recorded from the ES muscle of a representative participant. Traces show the average of 10 test MEPs (black traces) and conditioned MEPs (Cond. MEP, grey traces) indicated by arrows. (**B**) Group data showing SICI in the ES muscle (n = 7). The abscissa shows the time points measured (baseline, 10 min and 20 min after 30 min of unilateral arm cycling). The ordinate shows the size of the conditioned MEP expressed as a % of the test MEP). Note that SICI decreased (increased conditioned MEP size) at 10 and 20 min after cessation of the arm cycling. (**C**) Individual data. Note that majority of participants show decreases in SICI in the ES muscle at 20 min after cessation of the arm cycling compared with baseline. Error bars indicate the standard error of the mean (SEM). *p < 0.05, comparison between post-exercise and baseline.

To examine the contribution of the M1 to changes in ES MEPs, we employed a previously described method^27^ by testing short-interval intracortical inhibition (SICI) in 7 participants. SICI was performed prior to and following the arm cycling. Figure 3A illustrates averaged data from test and conditioned MEPs recorded from the ES muscle while testing SICI in a representative participant. Note that the magnitude of SICI in ES decreased following the arm cycling compared with baseline. There was an effect of SUB-TIME on SICI in ES (F_2,12_ = 5.73; p = 0.018; n = 7; Fig. 3B). *Post-hoc* tests showed that SICI was decreased at 10 min (66.51 ± 14.42%; p = 0.01), and at 20 min (66.68 ± 14.76%; p = 0.02) after the arm cycling compared with baseline (58.67 ± 12.67%). Note that majority of participants show reduced SICI in ES at 20 min after the arm cycling compared with baseline (Fig. 3C). There was no difference in root-mean-sqaure (rms) EMG in ES across time points (F_2,12_ = 1.13; p = 0.36).

### Cervicomedullary motor evoked potentials (CMEPs) in the ES muscle did not change after the arm cycling

**Figure 4:**
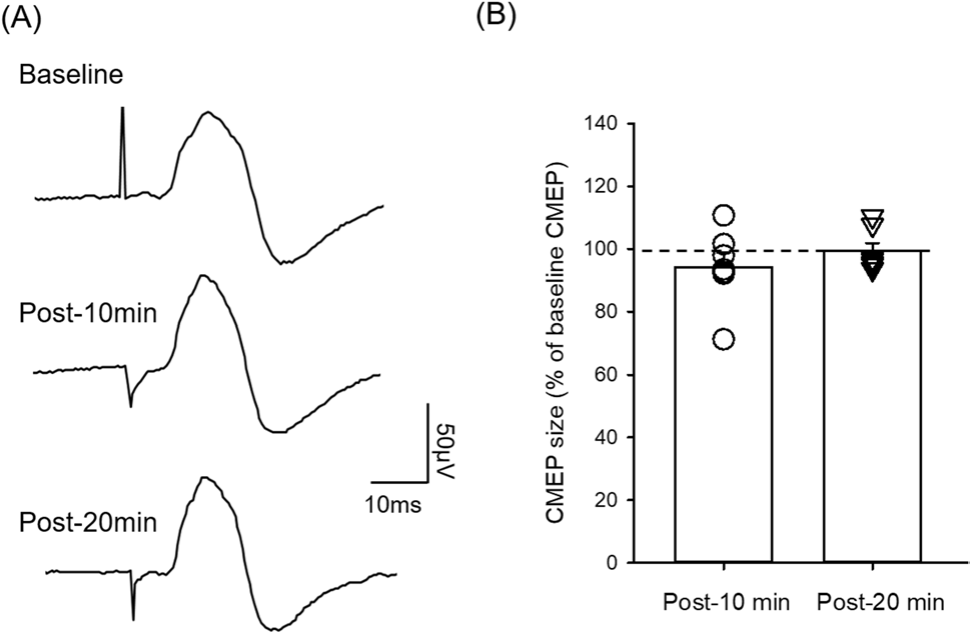
Cervicomedullary motor evoked potentials (CMEPs) in the erector spinae (ES) muscle. (**A**) CMEPs recorded from the ES muscle of a representative participant. Traces show the average of six test CMEPs before (baseline) and after 30 min of unilateral arm cycling. (**B**) Group data showing CMEPs in the ES muscle (n = 7). The abscissa shows the time points measured. The ordinate shows the size of the CMEPs (as a % of the baseline CMEP). The horizontal line represents the size of the CMEP at baseline. Individual data is shown for each time point. Error bars indicate the standard error of the mean (SEM).

To examine the contribution of spinal excitability to the changes in ES MEPs following the arm cycling, electrical stimulation was used at cervicomedullary junction to elicit cervicomedullary motor evoked potentials (CMEPs) in the ES (n = 7). Figure 4A illustrates traces of averaged CMEPs in the ES muscle from a representative participant. Note that the amplitudes of CMEP in the ES muscle remain the same after the arm cycling compared with baseline. The latency of CMEP was significantly shorter than MEPs elicited by TMS (CMEP: 8.03 ± 1.11 ms, MEP: 14.63 ± 1.97 ms; p < 0.001; n = 7), indicating that the stimulation activated corticospinal axons directly. There was no effect of SUB-TIME on the size of CMEP in the ES muscle (F_2,12_ = 1.40; p = 0.28; Fig. 4B), suggesting that the amplitude of the ES CMEPs remained the same after the arm cycling compared with baseline. There was no difference in amplitudes of rmsEMG in ES across all time points (F_2,12_ = 2.26; p = 0.15).

### Effects with tDCS

**Figure 5:**
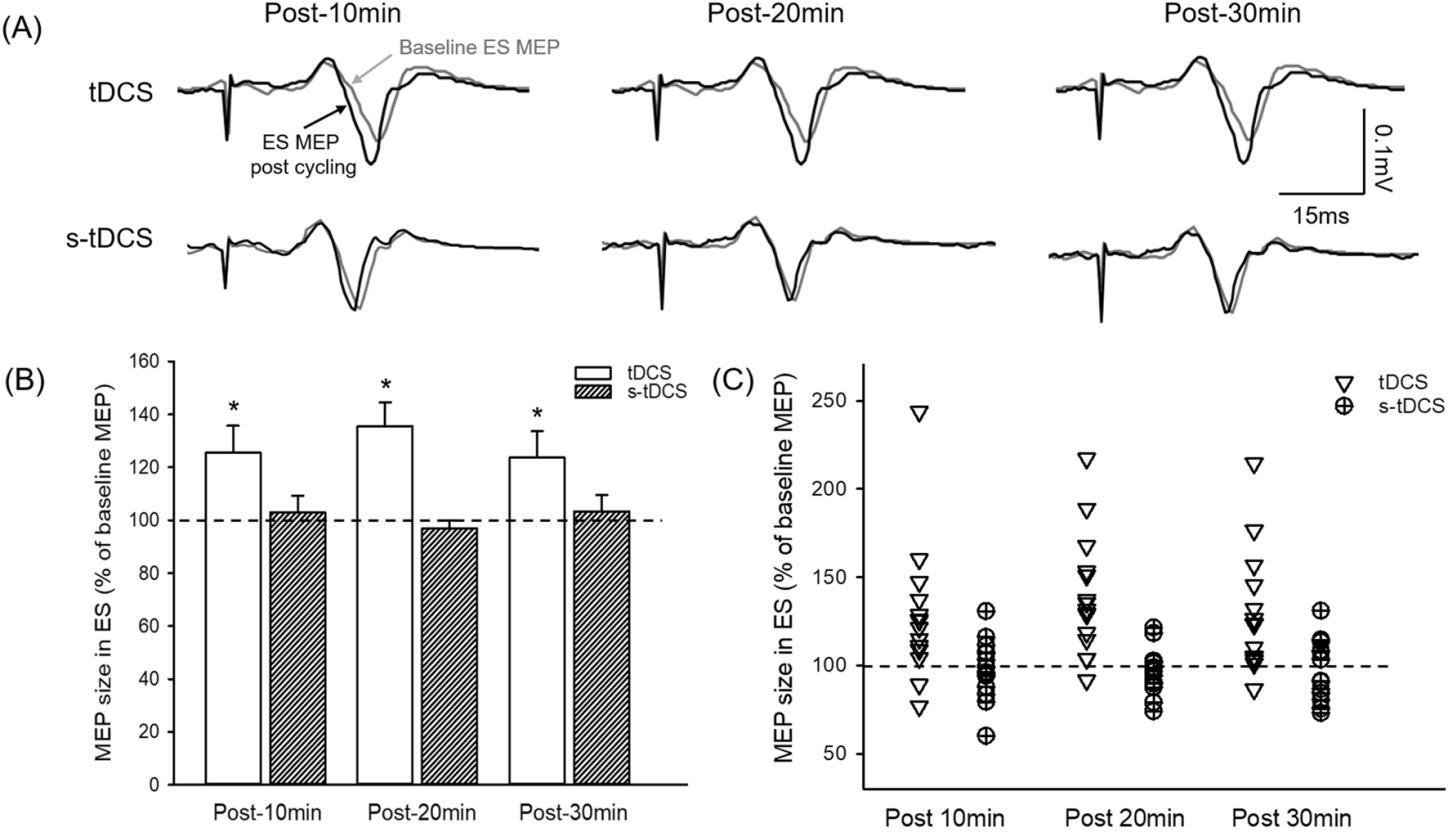
Effect of 20 min of unilateral arm cycling with bilateral transcranial direct current stimulation (tDCS) on motor evoked potentials (MEPs) in the erector spinae (ES) muscle. (**A**) MEP traces recorded from the ES muscle of a representative participant. Traces show the average of 10 MEPs in the ES muscle before (grey graces) and after (black traces) 20 min of unilateral arm cycling with bilateral tDCS or sham-tDCS (s-tDCS). (**B**) Group data (n = 15) showing ES MEPs after 20 min of unilateral arm cycling with bilateral tDCS (unfilled) and sham-tDCS (s-tDCS, pattern). The abscissa shows the time points measured after cessation of the arm cycling and the ordinate shows the size of ES MEP (as a % of baseline ES MEP). The horizontal dashed line represents the size of the ES MEP at baseline. Note that the size of ES MEP increases after the arm cycling in the bilateral tDCS condition but remains the same in the s-tDCS condition. (**C**) Individual data showing that majority of participants demonstrate increases in ES MEP after 20 min of arm cycling with bilateral tDCS. The increases in ES MEP are less clear in the s-tDCS condition. Error bars indicate the standard error of the mean (SEM). *p < 0.05, comparison between post-exercise and baseline.

Based on previous research suggesting that the cross-transfer effect reflects neural interactions of bilateral M1s^14^, bilateral tDCS with the anode over the M1 ipsilateral to the cycling arm and cathode over the opposite M1 was applied in conjunction with 20 min of the arm cycling. Figure 5A illustrates traces of averaged MEPs in the ES muscle from a representative participant. Note that the size of ES MEP increased after 20 min of combined tDCS and unilateral arm cycling, whereas the MEP amplitudes remained the same after the cycling with s-tDCS. Results showed an effect of CONDITION (F_1,14_ = 9.19; p = 0.009; n = 15), TIME (F_3,42_ = 4.53; p = 0.08), and their interaction (F_3,42_ = 4.23; p = 0.01) on the size of ES MEP (Fig. 5B). Note that majority of participants show increased ES MEP after the arm cycling with the tDCS compared with the arm cycling with s-tDCS (Fig. 5C). Post-hoc tests showed that in the combined tDCS and arm cycling condition the size of ES MEP increased at 10 min (125.46 ± 39.70%; p = 0.01), 20 min (135.44 ± 35.36%; p < 0.001), and 30 min (123.77 ± 39.06%; p = 0.02) after the cycling compared with baseline. Conversely, ES MEP remained the same across all time points in the s-tDCS with arm cycling condition. In addition, there was no effect of CONDITION (F_1,14_ = 0.10; p = 0.76), TIME (F_3,42_ = 1.54; p = 0.22), or their interaction (F_3,42_ = 0.88; p = 0.46) on background EMG in ES. Our results indicate that 20 min of combined arm cycling and tDCS increased corticospinal excitability of the ES muscles, while 20 min of unilateral arm cycling with s-tDCS did not change the excitability.

**Figure 6:**
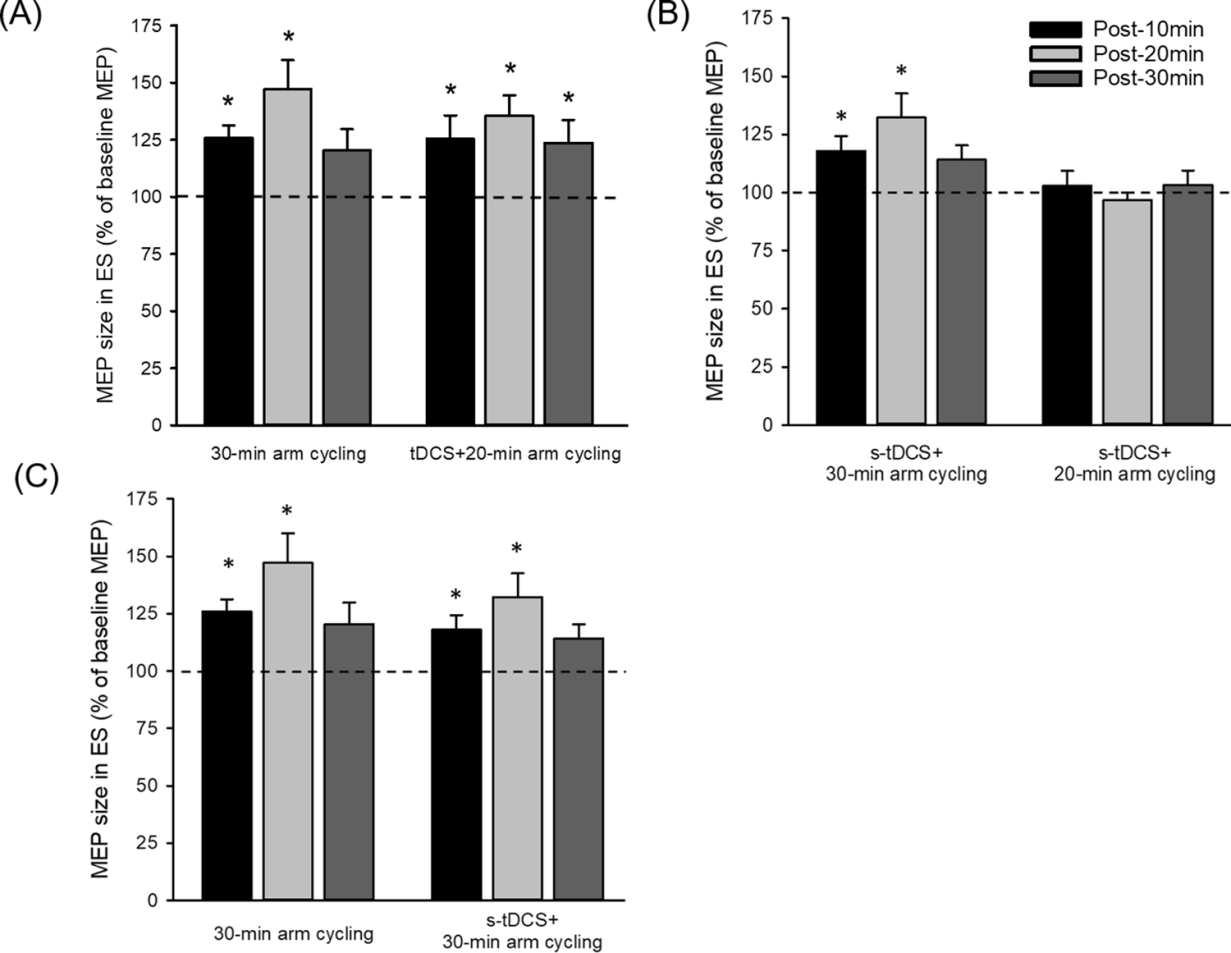
Effect of bilateral transcranial direct current stimulation (tDCS) and duration of the arm cycling on motor evoked potentials (MEPs) in the erector spinae (ES) muscle. (**A**) Group data showing increases in ES MEPs at 10 (black), 20 (light grey), and 30 min (dark grey) after cessation of unilateral arm cycling in conditions of 30 min of unilateral arm cycling (n = 15) and tDCS with 20 min of arm cycling (n = 15). Note that MEPs increase to the same extent in both conditions. (**B**) Group data showing increases in ES MEPs after 30 min of arm cycling with sham-tDCS (s-tDCS; n = 15), whereas no changes in ES MEPs after 20 min of arm cycling with s-tDCS (n = 15). (**C**) Group data (n = 15) showing increases in ES MEPs after 30 min of the arm cycling alone and with s-tDCS. Note that ES MEPs increase to the same extent in both conditions. The abscissa shows the time points measured after cessation of the arm cycling and the ordinate shows the size of ES MEP (as a % of baseline ES MEP). The horizontal dashed line represents the size of the ES MEP at baseline. Error bars indicate the standard error of the mean (SEM). *p < 0.05, comparison between post-exercise and baseline.

We compared the size of ES MEP obtained from participants who underwent 20 min of combined arm cycling and tDCS (n = 15) with those undergoing 30 min of arm cycling alone (n = 15). Results revealed an effect of TIME (F_3,84_ = 11.04; p < 0.001; Fig. 6A) but no interaction between TIME and GROUP (F_3,84_ = 0.58; p = 0.63) on the size of ES MEP, suggesting that the MEPs increased to a similar extent in both groups after the arm cycling. There was no difference in normalised background EMG in ES between the two groups (F_3,84_ = 1.66; p = 0.18).

### Effects of duration of the arm cycling

We compared the size of ES MEP obtained from the participants who underwent 30 min of arm cycling with s-tDCS (n = 15) with those undergoing 20 min of arm cycling with s-tDCS (n = 15). We found an effect of TIME (F_3,84_ = 3.60; p = 0.017) and an interaction between TIME and GROUP (30 min vs. 20 min of arm cycling; F_3,84_ = 4.85; p = 0.004) on the size of MEP in the ES muscle. Post-hoc tests showed that the amplitude of ES MEP increased at 10 min (121.46 ± 20.96%; p = 0.04), and at 20 min (135.28 ± 39.36%; p < 0.001) after 30 min of arm cycling (Fig. 6B). However, the size of ES MEP remained the same after 20 min of arm cycling compared with baseline (F_3,42_ = 0.52; p = 0.67). There was no difference in normalised background EMG in ES between conditions or across time points (all p > 0.05). These findings indicate 30 min of arm cycling increased corticospinal excitability of the ES muscle, whereas 20 min of arm cycling did not. In addition, we observed the same amount of increases in ES MEP between 30 min of arm cycling alone and 30 min of arm cycling with s-tDCS (F_1,14_ = 0.19; p = 0.67; Fig. 6C), indicating that s-tDCS did not influence the effect of unilateral arm cycling on facilitation of the ES MEPs.

### Functional outcomes

There was no difference in the rmsEMG of ES MVCs before and after 30 min of arm cycling (t = 1.14; p = 0.27; n = 15). For the rapid shoulder flexion task, there was no difference in the reaction time (t = 0.76; p = 0.46), EMG onset of ES (t = 0.79; p = 0.44), or EMG onset of ES with respect to AD (t = 0.49; p = 0.63) prior to and following 30 min of arm cycling.

## Discussion

Our findings demonstrate that 30 min of unilateral arm cycling can increase corticospinal drive to a trunk muscle in healthy young adults and the effect outlasts after cessation of the cycling. We found that SICI decreases and CMEPs remain the same after the arm cycling, suggesting that the crossed corticospinal facilitation of the trunk muscle induced by unilateral arm cycling is mediated, at least in part, at the cortical level. These findings were confirmed by our results which demonstrated 20 min of unilateral arm cycling with tDCS facilitates corticospinal excitability of the ES muscle to a similar extent as 30 min of unilateral arm cycling alone. There was no after-effect observed after 20 min of unilateral arm cycling with s-tDCS, highlighting the importance of duration of the arm cycling to induce short-term plasticity in corticospinal pathways projecting to the trunk muscle.

It is well documented that training with one limb increases strength and performance of the contralateral, untrained limb, so called cross-transfer effect. Many studies have shown that the MEP size in a relaxed, untrained muscle increases during unilateral tonic contractions^21,28,29^ as well as after unilateral training with the contralateral muscle of the opposite limb^10,13,30,31^. Here we extended these results and for the first time examine the effect of rhythmic arm movement on facilitation of corticospinal pathways projecting to the ES muscle and the mechanisms underlying this effect. In our study, corticospinal excitability was measured from the ES muscle which was not a primary muscle carrying out the arm cycling. We found that the size of ES MEPs increases following 30 min of unilateral arm cycling, with the facilitatory effect outlasting for up to 20 min. Our results agree with previous studies demonstrating that the size of ES MEP increased during tonic contractions of upper-limb muscles^18–20^. Our findings are also in agreement with evidence showing that a single session of repetitive upper-limb movements (e.g. wrist flexion) can induce acute neural plasticity in corticospinal tract projections to the contralateral, relaxed muscle^13,31,32^. We suggest that it is possible to induce short-term plasticity in the corticospinal projections to the trunk muscles by the use of upper limbs.

We found a decrease in intracortical inhibition in the ES muscle after the arm cycling. This result is consistent with several lines of evidence suggesting that intracortical circuits contribute to the neural adaptations within the relaxed, untrained muscle^30,33^. The finding is also in keeping with previous studies showing that using repetitive TMS over the M1 contralateral to the untrained muscle can modulate the effect of cross-transfer gained in the untrained muscle^10,32^, highlighting motor cortical involvement in the cross-transfer effect observed in the untrained muscle. Corticospinal neurons arising within the M1 project to arm muscles through dorsolateral column of the spinal cord^34,35^. It is therefore possible that intraspinal branching of corticospinal axons contribute to the facilitatory effect of the ES muscle. Indeed, plasticity of spinal cord circuits during and after cessation of the movement is not isolated to the moving muscle^36^. For example, rhythmic bilateral arm cycling reduces the amplitude of Hoffmann (H-) reflex in soleus muscles^37,38^ and the effect outlasts the duration of activity^39^. Evidence has also shown subcortical mechanisms contribute to crossed facilitation between upper-limbs in humans^29,40^. The facilitation of the ES MEP after the arm cycling observed in our study could be the result of modulation of spinal cord circuits. Since we did not observe changes in the amplitudes of CMEPs in the ES muscle after the arm cycling, it is unlikely that our results are influenced by changes in corticospinal transmission or motoneuron excitability^41,42^. Only few studies measured H-reflex in the untrained muscles following unilateral strength training^43–45^ and all of the studies reported no change in H-reflex amplitude after the unilateral strength training; changes in the H-reflex amplitudes were predominantly observed in the trained muscle^43–45^. Our results are also consistent with previous studies showing the minimal contribution of subcortical pathways to crossed facilitation between limb muscles as well as between arm and trunk muscles when similar low levels of voluntary contractions were performed^19,40^.

Another interesting finding is that combined bilateral tDCS with 20 min of arm cycling increases the facilitatory effect on corticospinal excitability of the ES muscle to the same extent as with 30 min of arm cycling alone. However, the size of ES MEP did not increase after 20 min of arm cycling with sham-tDCS. Our novel findings suggest that bilateral tDCS increases crossed corticospinal facilitation between arm and trunk muscles, advancing acute changes in the corticospinal drive to the trunk muscles. This is in agreement with a recent study showing that tDCS could prolong the cross-transfer effect of the unilateral training in the untrained muscle^17^. Combined tDCS with unilateral strength training has been shown to result in a superior effect on facilitation of corticospinal projections to the untrained muscle compared with unilateral strength training alone for the same amount of time^16,17,46^. It is suggested that the effects of tDCS are due to changes in membrane excitability of the cortical tissues underlying the stimulation, and modulations of synaptic efficacy connecting between motor cortical neurons^47–49^. Its aftereffects are polarity-specific, with anodal and cathodal tDCS inducing facilitatory and inhibitory effects on the stimulated cortical region, respectively. Studies using rTMS or tDCS over the untrained M1 have shown modulation of the cross-transfer effect in the untrained muscle^10,32^. Evidence has also shown reduced interhemispheric inhibition from the trained M1to the untrained M1, together with decreased intracortical inhibition in the untrained M1 after the unilateral strength training^15^, suggesting the contribution of transcallosal inhibition to the cross-transfer effect. Although mechanisms underlying bilateral tDCS remains unclear, some studies propose its effect resides in modulation of interhemispheric interactions^50–53^. The electrode montage chosen in our study aimed to increase excitability of the M1 ipsilateral to the cycling arm and, at the same time, reduce inhibition from the opposite M1. Based on our findings that intracortical inhibition was reduced after unilateral arm cycling, we speculate that the effect of bilateral tDCS on crossed facilitation between arm and trunk muscles is due to facilitation of the M1 ipsilateral to the cycling arm, thereby increased corticospinal projections to the contralateral muscles. Our speculation is supported by a recent study showing increased corticospinal excitability and reduced SICI in the untrained muscle after bilateral tDCS with unilateral motor training^16^.

Furthermore, we found the duration of unilateral arm cycling contributing to the short-term neural plasticity in the corticospinal drive to the trunk muscles. Our results revealed that 20 min of arm cycling with sham-tDCS did not facilitate corticospinal excitability of the ES muscle, whereas 30 min of arm cycling with sham-tDCS did and the facilitatory effect was to the same extent as 30 min of arm cycling alone. Although the facilitatory effect was observed in the untrained muscle when the training duration was less than 30 min^13,54^, the context of the training protocols involved strong voluntary muscle contractions, i.e. strength training, or motor learning and skill acquisition. Evidence has suggested that the level of contractions and types of motor training can influence the amount of crossed facilitation observed in the untrained muscle^21,55^. To the best of our knowledge, this is the first study reporting that unilateral arm cycling alone for 20 min or less is unlikely to induce acute changes in corticospinal drive to the trunk muscle. Although we did not test the minimum duration of the unilateral arm cycling required for inducing corticospinal facilitation of the trunk muscle, our results highlight the importance of training duration influencing crossed corticospinal facilitation between arm and trunk muscles.

While previous studies report improved motor performance in the untrained muscle after unilateral motor training, we did not observe changes in either APAs or MVCs of the ES muscle after the unilateral arm cycling. Note that majority of studies reporting changes in performance employed protocols of muscle strengthening which involved a higher level of voluntary contractions of the trained muscles. Several lines of evidence have demonstrated that higher levels of voluntary contractions induce greater corticospinal facilitation in the untrained muscle^21^. Since the level of voluntary contractions of arm muscles required in the arm cycling (less than ~ 20%MVC) was lower than that required in the strength training (70–80%MVC)^17,30^, lack of improvement in trunk motor function could be due to different levels of muscle contractions applied. Other studies reported improved movement acceleration in the untrained muscle after unilateral motor practice, such as a ballistic index finger abduction and repetitive wrist flexion–extension^9,10,13,31^. The context of motor learning influences the magnitude of cross-transfer effects. For instance, sequential learning does not induce the cross-transfer effect^56,57^, whereas a simple, ballistic movement does^13^. It has been suggested that the extent to which the degree of cross-transfer effect depends on the neural substrates supporting the learning of motor skills^22,58^.

Our findings are clinically relevant as they add to previous work showing that performance gained in the untrained muscle may be due to crossed corticospinal facilitation induced by the muscle actively involved in training^59,60^. Here we demonstrate, for the first time, that the use of the arms can induce acute neural plasticity in the physiological pathways projecting to the trunk muscles in healthy volunteers. The finding suggests the use of arm exercise to improve trunk motor function will be of use in patient populations. However, caution is required when interpreting clinical implications of our findings since the findings were from a single recording site (i.e. ES) and from a small number of healthy young adults. It is unclear whether the effects observed in this study can be generalised to other muscles of the trunk, such as rectus abdominis, or patient populations. Furthermore, we did not examine the effects of multiple sessions of the arm cycling exercise in either healthy adults or patient populations in this study. Whether the cross-transfer effect observed in the ES muscle induced by the arm cycling exercise would be present and result in improvement in trunk function in patient populations warrants further investigation. Nevertheless, our results are in support of a recent study reporting improved static sitting balance in patients with incomplete spinal cord injury after 5 weeks of arm crank ergometer spin training^61^. Impaired trunk control is common in patients with neurological disorders, such as stroke and spinal cord injury, which subsequently hinders their ability to use their limbs in activities of daily living^23,24^. As such, the upper-limb exercise protocol used in our study potentially represents an opportunity for trunk rehabilitation in patient populations.

In conclusion, our findings demonstrate that 30 min of unilateral arm cycling increases corticospinal excitability of the ES muscle and the facilitatory effect outlasts for up to 20 min after cessation of the arm cycling. We suggest that intracortical mechanisms within the M1 may contribute to the facilitatory effect observed in the ES muscle. Furthermore, we show that this crossed facilitatory effect between arm and trunk muscles can be enhanced by bilateral tDCS, highlighting the notion that cortical mechanisms play a role in mediating the physiological interactions between arm and trunk muscles in healthy humans.

## Methods

### Participants

Thirty healthy adults (male: female 15: 15; 6 left-handed; mean [SD] age 21 [3] years) took part in the study. Participants were excluded if they had contraindications to TMS (i.e. metal implants, history of epilepsy or fits, previous brain injury, neurosurgery, actively taking antidepressant or other neuromodulatory drugs)^62^, or did not show visible MEPs elicited by TMS in the erector spinae (ES) muscle. Of 30 participants, 15 underwent 30 min of unilateral arm cycling, with and without a concurrent sham transcranial direct current stimulation (s-tDCS) on two different occasions (inter-test interval: 33 ± 22 days; range 6–70 days). The other 15 participants underwent 20 min of unilateral arm cycling with tDCS and s-tDCS. Bilateral tDCS electrode montage was used in which the anode was placed over the primary motor cortex (M1) contralateral to the recording ES muscle, and the cathode was placed over the opposite M1^50,63^. Those 15 participants were preselected from a total of 25 participants to ensure that they showed facilitation of amplitudes of ES MEPs following 20 min of tDCS alone, controlling for inter-subject variability of the tDCS effect^64,65^.

### Electromyography (EMG)

EMG recordings were obtained bilaterally from ES at the 12th thoracic vertebral level (T12) and anterior deltoid (AD) muscles, and unilaterally from biceps brachii (BB) and triceps brachii (TB) of the dominant arm in accordance with SENIAM guidelines (https://www.seniam.org/). Pairs of Ag/AgCl electrodes (self-adhesive, 2 cm diameter, CareFusion, UK) were positioned approximately parallel to the muscle fibre orientation. A ground electrode was placed at the 7th cervical vertebral level (C7). For ES, electrode bars were positioned 3 cm either side of the spinous processes. EMG data were filtered (10-500 Hz), amplified (1000 ×) and sampled at 1 kHz using a Micro 1401 data acquisition system and Signal v6.01or Spike v7 software (Cambridge Electronic Design [CED], UK) connected.

### Experimental procedures

A stationary ergometer fixed on a height-adjustable table was used for unilateral arm cycling in the study. Participants were seated upright and gripped the ergometer handle lightly with their dominant hand. The height and distance of the ergometer were adjusted individually so that all participants had their shoulder in line with the arm crank shaft, and reached to the maximal pedal distance with elbow extended, minimal movements of the shoulder (Fig. 1A). This position was determined to maximise activation of BB and TB during the arm cycling. Participants were instructed to perform 30 min of arm cycling against minimum resistance of the ergometer at 60 repetition per minute^66^. During the cycling, they were asked to maintain upright-seated posture without back support, and to minimise forward–backward movements of the trunk. Muscle activity of BB, TB and bilateral ES was recorded during unilateral arm cycling in the Spike software (Fig. 1B). To quantify the amount of activity of BB and TB used in the arm cycling, participants performed 2–3 brief (~ 2 s) maximal voluntary contractions (MVCs) of the elbow flexors and elbow extensors isometrically, with the shoulder at neutral position and the elbow flexed at 90°. Consistent verbal encouragement was given throughout. To examine the effect of the arm cycling on trunk motor function, MVCs of the ES and anticipatory postural adjustments (APAs) of the trunk were measured prior to and following the arm cycling. For the MVCs of the ES, participants performed 2 brief (~ 2 s) MVCs of the trunk extensors in a prone position with pelvis and legs fixed securely and resistance provided at the scapulae. To assess APAs of the trunk, a rapid shoulder flexion task was used^3^. Participants stood with feet in shoulder width, arms fully relaxed by the sides. They were asked to raise both arms to 90° as quickly as possible in response to a red LED light, located ~ 1 m away from them at eye level, for 10 times. The LED cue was proceeded by a verbal warning signal given by the experimenter. The interval between the verbal warning signal and the light flashing was randomised.

### Transcranial magnetic stimulation (TMS)

TMS pulses were delivered via a Magstim 2002 monophasic stimulator (Magstim Company) through a figure 8 coil (loop diameter: 10 cm) or a double-cone coil (loop diameter: 11 cm). For individuals in whom a visible MEP could not be elicited in the ES muscle by the figure 8 coil, the double-cone coil was used. Stimulation was applied over the M1 ipsilateral to the dominant arm to induce MEPs in the contralateral ES. The optimal position (hotspot) for the coil was identified by moving the coil in small increments in the area. The hotspot was defined as the point where the largest MEP was evoked in the contralateral ES muscle. The position of the hotspot was marked on the scalp to ensure consistent coil placement throughout the testing. The coil was held against the head by an investigator, with the figure 8 coil handle pointed posteriorly and oriented ~ 45° away from the midline to induce a current flow in the anteromedial direction. When the double-cone coil was used, the handle was pointing vertically upwards. The location of the hotspot with respect to the vertex was recorded to allow coil reposition to a similar location for the same participant between different testing sessions. Each participant had at least two visits on different occasions to complete the study. TMS measurements included MEPs and short-interval intracortical inhibition (SICI).

### MEP

Active motor threshold (AMT) of the ES was defined as the lowest intensity of TMS at which visible MEPs were evoked in at least three out of six consecutive stimuli (74.92 ± 12.52% of maximal stimulator output [MSO]). TMS intensity was determined to generate MEPs in ES at peak-to-peak amplitudes of ~ 0.1 mV (0.10 ± 0.07 mV)^19^. Single TMS pulses were delivered in 4 s intervals while participants were seated upright with arms relaxed by the sides. MEPs were tested prior to (baseline), and 10, 20, and 30 min after the arm cycling. Ten MEPs were averaged from each time point.

### SICI

To examine the contribution of the M1 to changes in ES MEPs, we employed a previously described method^27^ by testing short-interval intracortical inhibition (SICI) in 7 participants. SICI was performed prior to and following the arm cycling. A conditioning stimulus (CS) was set at an intensity required to elicit ~ 50% of SICI (57.14 ± 6.59% of MSO, corresponding to ~ 70% of AMT). A test stimulus (TS) was set at an intensity that elicited an MEP with a peak-to-peak amplitude of ~ 0.1 mV (89.42 ± 10.05% of MSO). The CS was delivered 2.5 ms before the TS. A set of 20 stimuli (10 test MEPs and 10 conditioned MEPs) was applied in a computer-generated random order. SICI was calculated by expressing the conditioned MEP peak-to-peak amplitude as a percentage of the test MEP peak-to-peak amplitude.

### Cervicomedullary motor evoked potential (CMEP)

To examine the contribution of spinal excitability to the changes in ES MEPs following the arm cycling, electrical stimulation was used at cervicomedullary junction to elicit cervicomedullary motor evoked potentials (CMEPs) in the ES (n = 7). A high-voltage electrical current (100 μs duration, Digitimer DS7AH) was passed between adhesive Ag–AgCl electrodes fixed to the skin behind the mastoid process^42,67^. When the optimal position of electrodes (position that elicited the largest CMEPs) was identified, the electrodes were secured in place with tape to ensure that they remain at the same place throughout the experiment. Stimulus intensity was set to generate a consistent CMEP in ES within tolerance of the participants (238.57 ± 13.45 mA; amplitude of CMEP: 0.05 ± 0.003 mV). The latency of CMEP was monitored frame-by-frame to ensure the accuracy and consistency of the responses (averaged latency of CMEP: 8.03 ± 1.11 ms). CMEPs were tested prior to and following the arm cycling. Five to ten stimuli were delivered at each time point.

### Transcranial direct current stimulation (tDCS)

To examine if the facilitatory effect of the arm cycling on the ES muscle can be improved by increasing excitability of the M1, 15 participants underwent a shorter version of the unilateral arm cycling with dual-hemispheric (bilateral) tDCS. A pair of rubber electrodes (5 × 5 cm^2^) were placed over bilateral M1. The side of the electrodes attaching to the scalp was covered by a thin layer of conductor transmission gel to reduce impedance. Care was taken to ensure that the gel was limited to only under the electrodes. The anode was placed on the ES hotspot ipsilateral to the dominant arm (the cycling arm); the cathode was placed symmetrically over the vertex on the opposite M1. The electrodes were secured firmly on the head using a velcro-strap or with tape to ensure that they remain at the same place throughout the arm cycling. The electrical current of 2 mA was applied (neuroConn DC-Stimulator Plus, neuroConn GmbH, Ilmenau, Germany) during 20 min of arm cycling, with 30 s ramp-up and 30 s ramp-down. The same participants also underwent 20 min of arm cycling with sham-tDCS applied, where the electrical stimulation was on for only the first 30 s to provide tingling sensation on the scalp. Two sessions were at least 72 h apart for all participants (12.4 ± 11.5 days). The order of the sessions was randomised using an online randomiser (www.random.org). Participants were blinded to the conditions and were asked to indicate whether the condition was real or sham-tDCS at the end of the stimulation (tDCS: 14 out of 15 identified; s-tDCS: 3 out of 15 identified; Chi-squared = 0.27; p = 0.61), suggesting that the participants were blinded. Further, to examine if duration of the unilateral arm cycling influenced the facilitatory effect of the ES muscle, a combined s-tDCS and 30 min of unilateral arm cycling carried out in a different cohort (n = 15). The same procedure for obtaining MEPs in the ES muscle was applied.

### Data analysis

Peak-to-peak amplitudes of average MEPs obtained from each time point were measured from ES EMG recordings and expressed as a percentage of the baseline MEP amplitude. Pre-stimulus EMG obtained from ES was calculated as root-mean-square EMG (rmsEMG) amplitudes in a 100 ms window prior to the stimulus and presented as a percentage of ES MVC. MVCs of ES, BB and TB were calculated as rmsEMG amplitudes in a 500 ms window. EMG traces obtained from the rapid shoulder flexion task were rectified. The onset time of EMG activity in ES and AD for each frame was calculated as the time at which EMG activity exceeded 3 SD above the mean EMG level prior to the LED light^3,19,68^.

### Statistical analysis

Normal distribution was tested by the Shapiro–Wilk test. When data were not normally distributed (p < 0.05), non-parametric tests were used for. The Mauchly test was used in repeated measures analysis of variance (ANOVA) to test sphericity. When sphericity could not be assumed, the Greenhouse–Geisser correction statistics was used. To investigate the effect of the unilateral arm cycling on corticospinal excitability of the ES, repeated measures ANOVA was performed to examine the effect of TIME (baseline, 10, 20, and 30 min) on MEP size, and rmsEMG in the ES muscle. Repeated measures ANOVA was used to determine the effect of SUB-TIME (baseline, 10, and 20 min) on SICI and CMEPs in the ES muscle. To reveal the effect of combined tDCS and arm cycling on corticospinal excitability of the ES, two-way repeated measures ANOVAs were applied to examine the effect of TIME and interaction between TIME and CONDITION (tDCS vs. s-tDCS) on MEP in the ES muscle. A mixed-model repeated measures ANOVA was employed to determine the effect of TIME and the interaction between TIME and GROUP (20 min of arm cycling + s-tDCS vs. 30 min of arm cycling + s-tDCS). Bonferroni post hoc tests were employed to test for significant comparisons. Significance was set at p < 0.05. Group data are presented as the mean ± SD in the text.

### Ethical approval

The study was approved by University of Birmingham Research Ethics Committee (ref. ERN_18-2077AP3A) and performed in accordance with the Declaration of Helsinki. Recruitment was carried out from students at the corresponding author’s institution. All participants provided written informed consent.

## Data Availability

The data that support the findings of this study are available on request from the corresponding author. The data are not publicly available due to privacy or ethical restrictions.

## References

[1] Aruin AS, Latash ML (1995). Directional specificity of postural muscles in feed-forward postural reactions during fast voluntary arm movements. Exp. Brain Res..

[2] Hodges PW, Richardson CA (1997). Relationship between limb movement speed and associated contraction of the trunk muscles. Ergonomics.

[3] Chiou SY, Gottardi SE, Hodges PW, Strutton PH (2016). Corticospinal excitability of trunk muscles during different postural tasks. PLoS ONE.

[4] Haruyama K, Kawakami M, Otsuka T (2017). Effect of core stability training on trunk function, standing balance, and mobility in stroke patients. Neurorehabil. Neural Repair.

[5] Khanafer S, Sveistrup H, Levin MF, Cressman EK (2019). Age differences in arm-trunk coordination during trunk-assisted reaching. Exp Brain Res.

[6] Shaikh T, Goussev V, Feldman AG, Levin MF (2014). Arm-trunk coordination for beyond-the-reach movements in adults with stroke. Neurorehabil. Neural Repair.

[7] Baldissera FG, Esposti R (2013). The role of anticipatory postural adjustments in interlimb coordination of coupled arm movements in the parasagittal plane: II Postural activities and coupling coordination during cyclic flexion-extension arm movements, ISO- and ANTI-directionally coupled. Exp. Brain Res..

[8] Cetisli Korkmaz, N., Can Akman, T., Kilavuz Oren, G. & Bir, L. S. Trunk control: The essence for upper limb functionality in patients with multiple sclerosis. *Mult Scler Relat Disord* **24**, 101–106. doi:10.1016/j.msard.2018.06.013 (2018). 10.1016/j.msard.2018.06.013 29982105

[9] Carson RG, Kennedy NC, Linden MA, Britton L (2008). Muscle-specific variations in use-dependent crossed-facilitation of corticospinal pathways mediated by transcranial direct current (DC) stimulation. Neurosci. Lett..

[10] Lee M, Hinder MR, Gandevia SC, Carroll TJ (2010). The ipsilateral motor cortex contributes to cross-limb transfer of performance gains after ballistic motor practice. J. Physiol..

[11] Green LA, Gabriel DA (2018). The cross education of strength and skill following unilateral strength training in the upper and lower limbs. J. Neurophysiol..

[12] Barss TS, Klarner T, Pearcey GEP, Sun Y, Zehr EP (2018). Time course of interlimb strength transfer after unilateral handgrip training. J. Appl. Physiol..

[13] Carroll TJ, Lee M, Hsu M, Sayde J (2008). Unilateral practice of a ballistic movement causes bilateral increases in performance and corticospinal excitability. J. Appl. Physiol..

[14] Carson RG (2005). Neural pathways mediating bilateral interactions between the upper limbs. Brain Res. Brain Res. Rev..

[15] Hortobagyi T (2011). Interhemispheric plasticity in humans. Med. Sci. Sports Exerc..

[16] Kidgell DJ, Goodwill AM, Frazer AK, Daly RM (2013). Induction of cortical plasticity and improved motor performance following unilateral and bilateral transcranial direct current stimulation of the primary motor cortex. BMC Neurosci..

[17] Hendy AM, Teo WP, Kidgell DJ (2015). Anodal transcranial direct current stimulation prolongs the cross-education of strength and corticomotor plasticity. Med. Sci. Sports. Exerc.

[18] 18Davey, N. J., Lisle, R. M., Loxton-Edwards, B., Nowicky, A. V. & McGregor, A. H. Activation of back muscles during voluntary abduction of the contralateral arm in humans. *Spine (Phila Pa 1976)* **27**, 1355–1360. doi:10.1097/00007632-200206150-00019 (2002). 10.1097/00007632-200206150-00019 12065986

[19] Chiou SY, Strutton PH, Perez MA (2018). Crossed corticospinal facilitation between arm and trunk muscles in humans. J. Neurophysiol..

[20] Sasaki A, Milosevic M, Sekiguchi H, Nakazawa K (2018). Evidence for existence of trunk-limb neural interaction in the corticospinal pathway. Neurosci. Lett..

[21] Perez MA, Cohen LG (2008). Mechanisms underlying functional changes in the primary motor cortex ipsilateral to an active hand. J. Neurosci..

[22] Perez MA (2007). Neural substrates of intermanual transfer of a newly acquired motor skill. Curr. Biol..

[23] Verheyden G (2006). Trunk performance after stroke and the relationship with balance, gait and functional ability. Clin. Rehabil..

[24] Field-Fote EC, Ray SS (2010). Seated reach distance and trunk excursion accurately reflect dynamic postural control in individuals with motor-incomplete spinal cord injury. Spinal Cord.

[25] Jean-Charles L (2017). Interhemispheric interactions between trunk muscle representations of the primary motor cortex. J. Neurophysiol..

[26] Chiou SY, Hurry M, Reed T, Quek JX, Strutton PH (2018). Cortical contributions to anticipatory postural adjustments in the trunk. J. Physiol..

[27] Kujirai T (1993). Corticocortical inhibition in human motor cortex. J. Physiol..

[28] Chiou SY (2013). Co-activation of primary motor cortex ipsilateral to muscles contracting in a unilateral motor task. Clin. Neurophysiol..

[29] Muellbacher W, Facchini S, Boroojerdi B, Hallett M (2000). Changes in motor cortex excitability during ipsilateral hand muscle activation in humans. Clin. Neurophysiol..

[30] Kidgell DJ (2015). Increased cross-education of muscle strength and reduced corticospinal inhibition following eccentric strength training. Neuroscience.

[31] Carson RG (2004). Excitability changes in human forearm corticospinal projections and spinal reflex pathways during rhythmic voluntary movement of the opposite limb. J. Physiol..

[32] Stockel T, Carroll TJ, Summers JJ, Hinder MR (2016). Motor learning and cross-limb transfer rely upon distinct neural adaptation processes. J. Neurophysiol..

[33] Leung M, Rantalainen T, Teo WP, Kidgell D (2015). Motor cortex excitability is not differentially modulated following skill and strength training. Neuroscience.

[34] Alawieh A, Tomlinson S, Adkins D, Kautz S, Feng W (2017). Preclinical and clinical evidence on ipsilateral corticospinal projections: implication for motor recovery. Transl. Stroke Res..

[35] McKiernan BJ, Marcario JK, Karrer JH, Cheney PD (1998). Corticomotoneuronal postspike effects in shoulder, elbow, wrist, digit, and intrinsic hand muscles during a reach and prehension task. J. Neurophysiol..

[36] Brooke JD (1997). Sensori-sensory afferent conditioning with leg movement: gain control in spinal reflex and ascending paths. Prog. Neurobiol..

[37] Frigon A, Collins DF, Zehr EP (2004). Effect of rhythmic arm movement on reflexes in the legs: modulation of soleus H-reflexes and somatosensory conditioning. J. Neurophysiol..

[38] Zehr EP, Duysens J (2004). Regulation of arm and leg movement during human locomotion. Neuroscientist.

[39] Javan B, Zehr EP (2008). Short-term plasticity of spinal reflex excitability induced by rhythmic arm movement. J. Neurophysiol..

[40] Stedman A, Davey NJ, Ellaway PH (1998). Facilitation of human first dorsal interosseous muscle responses to transcranial magnetic stimulation during voluntary contraction of the contralateral homonymous muscle. Muscle Nerve.

[41] Ugawa Y, Uesaka Y, Terao Y, Hanajima R, Kanazawa I (1994). Magnetic stimulation of corticospinal pathways at the foramen magnum level in humans. Ann. Neurol..

[42] Taylor JL, Gandevia SC (2004). Noninvasive stimulation of the human corticospinal tract. J. Appl. Physiol..

[43] Dragert K, Zehr EP (2013). High-intensity unilateral dorsiflexor resistance training results in bilateral neuromuscular plasticity after stroke. Exp Brain Res.

[44] Fimland MS (2009). Neural adaptations underlying cross-education after unilateral strength training. Eur. J. Appl. Physiol..

[45] Lagerquist O, Zehr EP, Docherty D (2006). Increased spinal reflex excitability is not associated with neural plasticity underlying the cross-education effect. J. Appl. Physiol..

[46] Goodwill AM, Daly RM, Kidgell DJ (2015). The effects of anodal-tDCS on cross-limb transfer in older adults. Clin. Neurophysiol..

[47] Nitsche MA, Paulus W (2000). Excitability changes induced in the human motor cortex by weak transcranial direct current stimulation. J. Physiol..

[48] Nitsche MA (2003). Pharmacological modulation of cortical excitability shifts induced by transcranial direct current stimulation in humans. J. Physiol..

[49] Liebetanz D, Nitsche MA, Tergau F, Paulus W (2002). Pharmacological approach to the mechanisms of transcranial DC-stimulation-induced after-effects of human motor cortex excitability. Brain.

[50] Tazoe T, Endoh T, Kitamura T, Ogata T (2014). Polarity specific effects of transcranial direct current stimulation on interhemispheric inhibition. PLoS ONE.

[51] Vines BW, Nair D, Schlaug G (2008). Modulating activity in the motor cortex affects performance for the two hands differently depending upon which hemisphere is stimulated. Eur. J. Neurosci..

[52] Williams JA, Pascual-Leone A, Fregni F (2010). Interhemispheric modulation induced by cortical stimulation and motor training. Phys. Ther..

[53] Waters-Metenier S, Husain M, Wiestler T, Diedrichsen J (2014). Bihemispheric transcranial direct current stimulation enhances effector-independent representations of motor synergy and sequence learning. J. Neurosci..

[54] Hendy AM, Kidgell DJ (2014). Anodal-tDCS applied during unilateral strength training increases strength and corticospinal excitability in the untrained homologous muscle. Exp. Brain Res..

[55] Leung M, Rantalainen T, Teo WP, Kidgell D (2018). The ipsilateral corticospinal responses to cross-education are dependent upon the motor-training intervention. Exp. Brain Res..

[56] Pascual-Leone A (1995). Modulation of muscle responses evoked by transcranial magnetic stimulation during the acquisition of new fine motor skills. J. Neurophysiol..

[57] Perez MA, Wise SP, Willingham DT, Cohen LG (2007). Neurophysiological mechanisms involved in transfer of procedural knowledge. J. Neurosci..

[58] Imamizu H, Shimojo S (1995). The locus of visual-motor learning at the task or manipulator level: implications from intermanual transfer. J. Exp. Psychol. Hum. Percept. Perform..

[59] Hamzei F (2012). Functional plasticity induced by mirror training: the mirror as the element connecting both hands to one hemisphere. Neurorehabil. Neural. Repair.

[60] Kowalczewski J, Chong SL, Galea M, Prochazka A (2011). In-home tele-rehabilitation improves tetraplegic hand function. Neurorehabil Neural Repair.

[61] Williams AMM (2020). Arm crank ergometer "spin" training improves seated balance and aerobic capacity in people with spinal cord injury. Scand J. Med. Sci. Sports.

[62] Rossi S, Hallett M, Rossini PM, Pascual-Leone A (2011). Screening questionnaire before TMS: an update. Clin. Neurophysiol..

[63] Mordillo-Mateos L (2012). Effects of simultaneous bilateral tDCS of the human motor cortex. Brain Stimul..

[64] Lee J (2019). Different brain connectivity between responders and nonresponders to dual-mode noninvasive brain stimulation over bilateral primary motor cortices in stroke patients. Neural Plast..

[65] Guerra A, Lopez-Alonso V, Cheeran B, Suppa A (2020). Variability in non-invasive brain stimulation studies: reasons and results. Neurosci. Lett..

[66] Forman DA, Philpott DT, Button DC, Power KE (2015). Cadence-dependent changes in corticospinal excitability of the biceps brachii during arm cycling. J. Neurophysiol..

[67] Benavides FD (2020). Cortical and subcortical effects of transcutaneous spinal cord stimulation in humans with tetraplegia. J. Neurosci..

[68] Hodges P, Cresswell A, Thorstensson A (1999). Preparatory trunk motion accompanies rapid upper limb movement. Exp. Brain Res..

